# An Overview of the Characteristics and Potential of *Calotropis procera* From Botanical, Ecological, and Economic Perspectives

**DOI:** 10.3389/fpls.2021.690806

**Published:** 2021-06-17

**Authors:** Amarpreet Kaur, Daizy R. Batish, Shalinder Kaur, Bhagirath S. Chauhan

**Affiliations:** ^1^Department of Botany, Panjab University, Chandigarh, India; ^2^Queensland Alliance for Agriculture and Food Innovation (QAAFI) and School of Agriculture and Food Sciences (SAFS), The University of Queensland, Gatton, QLD, Australia

**Keywords:** apple of sodom, calotrope, giant milkweed, physiological adaptations, phytochemistry, ethnomedicinal value, emerging invasive species

## Abstract

*Calotropis procera* (Aiton) Dryand. (commonly known as the apple of sodom, calotrope, and giant milkweed) is an evergreen, perennial shrub of the family Apocynaceae, mainly found in arid and semi-arid regions. It is a multipurpose plant, which can be utilized for medicine, fodder, and fuel purposes, timber and fiber production, phytoremediation, and synthesis of nanoparticles. It has been widely used in traditional medicinal systems across North Africa, Middle East Asia, and South-East Asia. At present, it is being extensively explored for its potential pharmacological applications. Several reports also suggest its prospects in the food, textile, and paper industries. Besides, *C*. *procera* has also been acknowledged as an ornamental species. High pharmacological potential and socio-economic value have led to the pantropical introduction of the plant. Morpho-physiological adaptations and the ability to tolerate various abiotic stresses enabled its naturalization beyond the introduced areas. Now, it is recognized as an obnoxious environmental weed in several parts of the world. Its unnatural expansion has been witnessed in the regions of South America, the Caribbean Islands, Australia, the Hawaiian Islands, Mexico, Seychelles, and several Pacific Islands. In Australia, nearly 3.7 million hectares of drier areas, including rangelands and Savannahs, have been invaded by the plant. In this review, multiple aspects of *C*. *procera* have been discussed including its general characteristics, current and potential uses, and invasive tendencies. The objectives of this review are a) to compile the information available in the literature on *C*. *procera*, to make it accessible for future research, b) to enlist together its potential applications being investigated in different fields, and c) to acknowledge *C*. *procera* as an emerging invasive species of arid and semi-arid regions.

## Introduction

*Calotropis procera* (Aiton) Dryand. is a soft-wooded, perennial shrub of the family Apocynaceae and subfamily Asclepiadaceae (the milkweed family). It is an evergreen xerophytic plant, generally found in arid and semi-arid habitats (Al-Rowaily et al., 2020). The word “*Calotropis*” is derived from Greek, meaning “beautiful,” which refers to its flowers; whereas “*procera*” is a Latin word referring to the cuticular wax present on its leaves and stem (Hassan et al., 2015). It is known by various common names such as apple of sodom, calotrope, giant milkweed, Indian milkweed, wild cotton, rubber tree, ushar, etc., in different parts of the world. Its subspecies, *C*. *procera* subsp. *procera* and *C*. *procera* subsp. *hamiltonii*, vary from each other in fruit morphology (Dhileepan, 2014). It also shares a close homology with its con-generic plant *C*. *gigantea* (CABI, 2021).

*Calotropis procera* is a multipurpose plant, which provides a wide range of provisioning ecosystem services. It has been widely used in traditional medicinal systems in North Africa, Middle East Asia, South Asia, and South-East Asia (Al Sulaibi et al., 2020). It has also been utilized for fiber, fuel, fodder, and timber purposes since antiquity (Batool et al., 2020). Owing to its socio-economic importance, it has been introduced in several parts of the world outside its native range (Asia and Africa). Morpho-physiological adaptations and the ability to tolerate a wide range of environmental conditions enabled its naturalization in the introduced habitats. Consequently, the plant has also been reported as an invasive weed of wastelands, overgrazed pastures, and poorly managed agricultural fields in several regions (CABI, 2021).

There is a plethora of literature available that demonstrates the pharmacological applications and economic importance of *C*. *procera*. However, very few studies have focused on general ecological and biological characteristics of the plant and its survival strategies under arid and semi-arid environments. Even fewer studies have addressed it as an invasive species and provided insights into its invasive abilities, potential distribution, and management options. In this review, multiple aspects of *C*. *procera* have been discussed to bring together the information available on the plant in the literature, identify its potential applications, acknowledge it as an emerging invasive species, and emphasize the knowledge gaps in ongoing research.

## Ecology and Biology

### Geographical Distribution, Macromorphology, and Reproductive Biology

**FIGURE 1:**
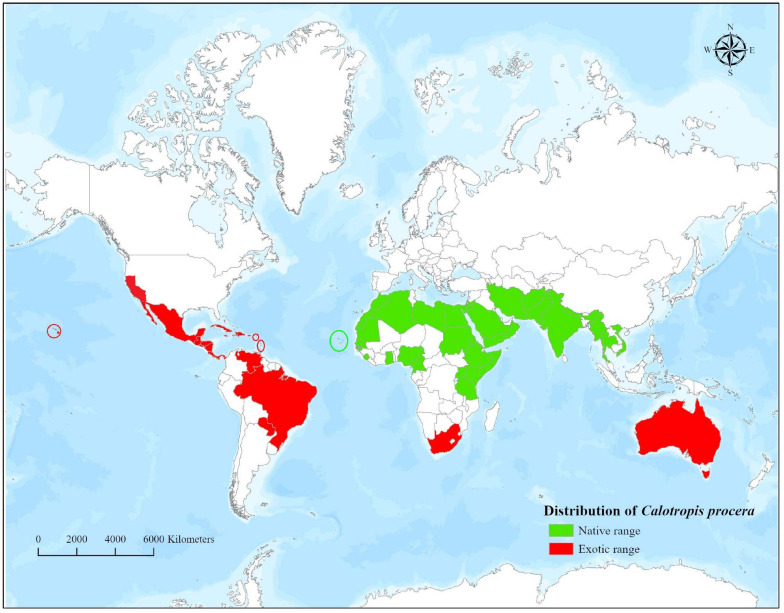
Worldwide distribution of *Calotropis procera.*

**FIGURE 2:**
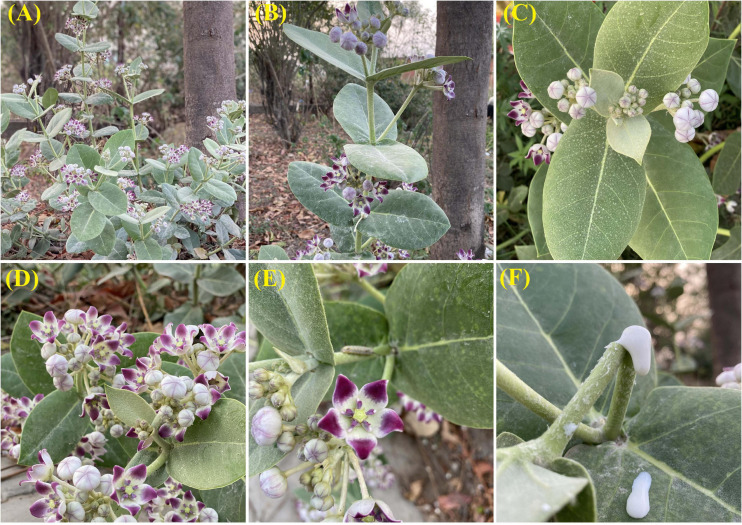
*Calotropis procera*: flowering plant **(A)**; phyllotaxy **(B)**; reproductive buds **(C)**; inflorescence **(D)**; individual flower **(E)**; and latex oozing out of the wounded stem **(F)**.

*Calotropis procera* is native to Africa, Arabian Peninsula, Western Asia, the Indian Subcontinent, and Indo-China (GRIN, 2021). However, the introduction of the plant outside its native boundaries has led to its naturalization in parts of Africa, Australia, and America (GRIN, 2021). The broad native and exotic geographical range of *C*. *procera* is presented in Figure 1. *Calotropis procera* is an evergreen shrub that may grow up to 6 m (usually 2.5–4 m) in height and has a deep taproot system (CABI, 2021; Figure 2). Young stems are grayish-green in color, smooth, and pubescent, whereas the mature stems have a deeply fissured bark (Hassan et al., 2015). The leaves are large, pale green, succulent, arranged in opposite phyllotaxy, and covered with cuticular wax (Batool et al., 2020; Figure 2). The plant contains a milky sap, which oozes out of any wound or injury in the aboveground parts (CABI, 2021; Figure 2).

**FIGURE 3:**
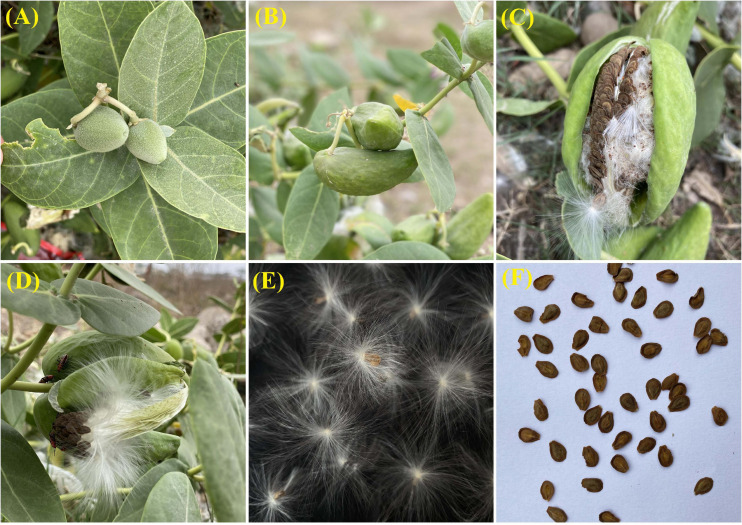
Fruit characteristics of *Calotropis procera*: immature fruits **(A)**; mature fruits **(B)**; dehisized fruits **(C,D)**; seeds with pappus **(E)**; seeds without pappus **(F)**.

Reproductive maturity in the plant is attained approximately 190 days after germination (Bebawi et al., 2015). Flowering takes place throughout the year, and pollination is carried out by insects, mostly bees and butterflies (Al Sulaibi et al., 2020; Batool et al., 2020). The inflorescence is dense and multiflowered umbellate cyme (3–15 flowers in a cluster; Figure 2), and the flowers are five-petaled, bisexual, sweet-smelling, and white in appearance with a characteristic purple tip (Al Sulaibi et al., 2020; Figure 2). Fruiting is limited to the warm months of the year when pollinators are the most abundant (Menge et al., 2017a). The fruits are ellipsoid or ovoid, containing 350–500 seeds with tufts of white, silky hair or pappus (Al Sulaibi et al., 2020; Figure 3). Seeds are generally disseminated by wind and water and occasionally, by birds and animals (Al Sulaibi et al., 2020). Seed longevity depends on several factors such as rainfall, soil moisture, seed burial depth, and soil type (Bebawi et al., 2015). Maximum seed germination (68–100%) occurs at 30°C and the maximum emergence (88%) is observed from a depth of 3 cm (Menge et al., 2016a). The plant also propagates through root suckers and regenerates through broken/cut stems and roots (Hassan et al., 2015).

### Stress Physiology

*Calotropis procera* has an exceptional ability to adapt and maintain productivity in severe arid conditions (Ramadan et al., 2014). It is a C_3_ plant that can survive drought, salinity, extreme temperatures, high vapor pressure deficit, and high photosynthetic active radiations (Frosi et al., 2013; Rivas et al., 2020). It can easily thrive in prolonged dry seasons with rainfall >150 mm per year (Dhileepan, 2014). The plant grows abundantly in xerophytic conditions on a variety of soils, without irrigation or application of fertilizers (Hassan et al., 2015). The plant has a great potential to endure stress caused by roadside pollutants and contaminated soils (Khalid et al., 2018; Ullah and Muhammad, 2020).

Plants surviving in the hostile environment of arid/semi-arid regions have advanced morpho-physiological adaptations and special defense mechanisms. So is the case of *C*. *procera*, in which multiple processes contribute to the resistance, resilience, and recovery of individuals growing under abiotic stress conditions (Rivas et al., 2017). The stems and leaves of *C*. *procera* are characterized by thick cuticle, lactiferous canals, and low specific leaf area (Tezara et al., 2011; Hassan et al., 2015). Leaves are found to be narrower and thicker under optimum moisture conditions, whereas they are broader and thinner under dry conditions (Pompelli et al., 2019). These factors help in the conservation of acquired resources and creating a water permeability barrier, thereby reducing the transpiration rate (Pompelli et al., 2019).

The plant also shows physiological and biochemical adaptations in terms of gas exchange and metabolic adjustments (Frosi et al., 2013). An efficient antioxidative system, leaf sugar dynamics, and photoprotective mechanisms guard the photosynthetic machinery of the plant under an extreme xerophytic environment (Rivas et al., 2017, 2020). Furthermore, the plant maintains a high photosynthetic rate despite reduced stomatal conductance, thus increasing water use efficiency, which is a fundamental characteristic for survival in arid and semi-arid ecosystems (Tezara et al., 2011; Frosi et al., 2013) as well as being able to quickly adjust the aquaporins of the root system when under salt stress (Coêlho et al., 2021). A metabolomic study revealed that *C*. *procera* rapidly adjusts the levels of soluble sugars, amino acids, triacylglycerols, and membrane lipids in response to water availability and water loss (Ramadan et al., 2014). Myo-inositol signaling is found to be induced in response to drought and salt stress in *C*. *procera* (Mutwakil et al., 2017).

Endophytic microbes such as *Pseudomonas stutzeri* and *Virgibacillus koreensis* are reported to be associated with *C*. *procera* under salt-stressed conditions, which may facilitate its survival under harsh conditions (Al-Quwaie, 2020). Similarly, endophytic fungal species, *Phaeoramularia calotropidis*, *Guignardia bidwellii*, *Curvularia hawaiiensis*, *Cochliobolus hawaiiensis*, *Alternaria alternata*, *Mucor circinelloides*, *Aspergillus* spp., *Penicillium* spp., *Fusarium* spp., *Chaetomium* spp., and *Candida* spp. are isolated from *C*. *procera*, which protects the plant from pests, pathogens, and herbivores (Nascimento et al., 2015; Rani et al., 2017).

## Phytochemistry

### Metabolic Profile

Several researchers have reported the presence of metabolites such as flavonoids, tannins, terpenoids, saponins, alkaloids, steroids, and cardiac glycosides in various parts of the plant (Mossa et al., 1991; Moustafa et al., 2010; Al-Rowaily et al., 2020). A list of secondary metabolites reported from the plant has been provided in Table 1.

The major phytochemical groups reported in the leaf extracts of *C*. *procera* are fatty acid ethyl esters (21.4%), palmitic acid esters (10.2%), linoleic acids (7.4%), and amino acids (8.1%) (Pattnaik et al., 2017). High-Performance Liquid Chromatography (HPLC) analysis of the leaves and bark ascertained the presence of total phenolic content (20.41–100.18 gallic acid equivalent mg g^–1^ dry weight), total flavonoid content (IC_50_ 18.33–92.92 catechin equivalent mg g^–1^ dry weight), sinapic acid (17.3 ± 2.11 to 9586.44 ± 0.78 mg kg^–1^), vanillic acid (9.43 ± 0.21 to 5051.7 ± 18.47 mg kg^–1^) and protocatechuic acid (2.46 ± 0.40 to 139.05 ± 1.37 mg kg^–1^) (Mehmood et al., 2020). The ratio of phenolic compounds and terpenoids was higher in leaves and lower in the case of root-bark of the plant (Kinda et al., 2020). Cardenolide-type terpenoids are mainly responsible for the phytotherapeutic abilities of the root-bark of *C*. *procera* (Kinda et al., 2020).

A total of 80% of the laticifer fluid of *C*. *procera* corresponds to rubber and the rest 20% is rich in basic proteins (anti-oxidant enzymes, cysteine proteases, tryptophan, etc.) with molecular masses in the range of 5–95 kilodaltons (Freitas et al., 2007; Das et al., 2011). A recent study deduced amino acid sequences of five previously identified cysteine peptidases from the latex of *C*. *procera* (procerain, procerain B, CpCP1, CpCP2, and CpCP3) (Freitas et al., 2020). These possess similar biochemical characteristics and high sequence homology with several other papain-like cysteine peptidases (Freitas et al., 2020). The presence of nearly 15 chitinase isoforms has also been reported in the latex of *C*. *procera* (Freitas et al., 2016).

The chemical profile of the essential oil of *C*. *procera* procured from Saudi Arabia and Egypt showed the presence of 90 compounds, of which terpenes (sesquiterpenes and diterpenes) were the main constituents along with hydrocarbons, aromatics, and carotenoids (Al-Rowaily et al., 2020). Hinesol, *trans*-chrysanthenyl acetate, 1,4-*trans*-1,7-*cis*-acorenone, phytol, myristicin, *n*-docosane, linoleic acid, *n*-pentacosane, and bicyclogermacrene represented the main compounds of essential oil (Al-Rowaily et al., 2020).

### Cytotoxicity and Phytotoxicity

*Calotropis procera* causes acute toxicity in various plant and animal cells, including human beings. Different plant parts, particularly the latex, are therefore tested against various cancer cell lines (Ibrahim et al., 2015; Viana et al., 2017; Al-Qahtani et al., 2020). Similarly, antibacterial and antihelminitic potential of the plant is being utilized in pharmacology (details provided in section “Pharmacological Applications”). However, the toxicity-bioactivity relationship of *C*. *procera* is still not well investigated. A few studies suggested that the plant induces acute cardiotoxicity and hepatotoxicity (de Lima et al., 2011). On the other hand, a safety evaluation study by Mossa et al. (1991) revealed that the use of *C*. *procera* extract in single high doses (up to 3 g kg^–1^) is not toxic for guinea pigs until the treatment of >90 days is provided. In another study, latex proteins of the plant when administrated orally, had no adverse immunological reactions in mice even at 5,000 mg kg^–1^; but their intraperitoneal administration caused death after 1 h in response to a dose of 150 mg kg^–1^ (Bezerra et al., 2017). These toxic aspects are not extensively researched and more studies are required to validate the medicinal prospects of *C*. *procera*.

Apart from that, extracts of the plant also possess significant pesticidal and fungicidal properties. It has been observed that life-history traits of *Sitophilus oryzae* L. (Coleoptera: Curculionidae) and *Rhyzopertha dominica* Fabricius (Coleoptera: Bostrichidae) were modulated by leaf extracts, latex proteins, and flavonoids isolated from *C*. *procera* (Nenaah, 2013a). Whole-plant extracts of the plant caused mortality of larva, reduced the number of eggs, and inhibited the oviposition of *Rhipicephalus microplus* Canestrini (Ixodida: Ixodidae) (Khan et al., 2019). Cysteine peptidases and osmotin purified from the latex of *C*. *procera* promoted membrane permeability, leakage of cellular content, and induction of reactive oxygen species in *Fusarium* spp. (de Freitas et al., 2011; Freitas et al., 2020). Such studies implicate that plant has a potential to be utilized as bioinsecticide and biofungicide in agricultural and industrial practices.

**TABLE 1:** Metabolic profile of *Calotropis procera.*

**S. No.**	**Compounds**	**Plant parts**	**References**
**Cardenolides**			
1.	12β-Hydroxycoroglaucigenin	Latex	Mohamed et al., 2015
2.	15β-Hydroxy calactin	Latex	Mohamed et al., 2015
3.	15β-Hydroxy uscharin	Latex	Mohamed et al., 2015
4.	19-Dihydrocalotropagenin	Whole plant	Sweidan and Abu Zarga, 2015
5.	Afrogenin	Latex	Mohamed et al., 2015
6.	Afroside	Latex	Mohamed et al., 2015
7.	Calactin	Latex, Whole plant	Mohamed et al., 2015; Sweidan and Abu Zarga, 2015
8.	Calactoprocin	Latex	Mohamed et al., 2015
9.	Calotoxin	Root, Latex, Whole plant	Kakkar et al., 2012; Mohamed et al., 2015; Sweidan and Abu Zarga, 2015
10.	Calotropin	Whole plant	Sweidan and Abu Zarga, 2015
11.	Digitoxigenin	Root	Kakkar et al., 2012
12.	Digitoxin	Root	Kakkar et al., 2012
13.	Digoxigenin	Root	Kakkar et al., 2012
14.	Ischaridin	Whole plant	Sweidan and Abu Zarga, 2015
15.	Ischarin	Whole plant	Sweidan and Abu Zarga, 2015
16.	Procegenin A	Latex	Mohamed et al., 2015
17.	Procegenin B	Latex	Mohamed et al., 2015
18.	Proceragenin	Root	Kakkar et al., 2012
19.	Uscharin	Latex, Whole plant	Mohamed et al., 2015; Sweidan and Abu Zarga, 2015
20.	Uzarigenin	Whole plant	Sweidan and Abu Zarga, 2015
**Steroids**			
1.	3β,27-Dihydroxy-urs-18-en-13,28-olide	Latex	Chundattu et al., 2016
2.	Calotroposides H–N	Root bark	Ibrahim et al., 2015
3.	Cyclosadol	Root	Kakkar et al., 2012
4.	Multiflorenol	Root; Latex	Kakkar et al., 2012; Chundattu et al., 2016
5.	Procesterol	Root	Kakkar et al., 2012
6.	Stigmasterol	Root bark, Root; Latex	Ibrahim et al., 2012; Kakkar et al., 2012; Chundattu et al., 2016
7.	Urs-19(29)-en-3-yl acetate	Latex	Chundattu et al., 2016
8.	Urs-19(29)-en-3-β-ol	Latex	Chundattu et al., 2016
9.	β-Sitosterol	Latex, Root, Whole plant	Kakkar et al., 2012; Sweidan and Abu Zarga, 2015; Chundattu et al., 2016
10.	β-Sitosterol glucoside	Whole plant	Sweidan and Abu Zarga, 2015
**Terpenes**			
1.	Calotropenol	Root	Kakkar et al., 2012
2.	Calotropenyl acetate	Root; Whole plant	Kakkar et al., 2012; Sweidan and Abu Zarga, 2015
3.	Calotropfriedelenyl acetate	Root bark	Ansari and Ali, 2001
4.	Calotroprocerol A	Root bark	Ibrahim et al., 2012
5.	Calotroprocerone A	Root bark	Ibrahim et al., 2012
6.	Calotroproceryl acetate A	Root bark	Ibrahim et al., 2012
7.	Calotroproceryl acetate B	Root bark	Ibrahim et al., 2012
8.	Calotropursenyl acetate	Root bark	Ansari and Ali, 2001; Ibrahim et al., 2012
9.	Dihydrophytoyl tetraglucoside	Root	Mittal and Ali, 2015
10.	Phytyl iso-octyl ether	Root	Mittal and Ali, 2015
11.	Procerasesterterpenoyl triglucoside	Root	Mittal and Ali, 2015
12.	Pseudo-taraxasterol acetate	Root bark	Ibrahim et al., 2012
13.	Taraxasterol	Root bark	Ibrahim et al., 2012
14.	β-Sitostenone	Root	Kakkar et al., 2012
**Proteins and Enzymes**			
1.	CpCP-1	Latex	Ramos et al., 2013
2.	CpCP-2	Latex	Ramos et al., 2013
3.	CpCP-3	Latex	Ramos et al., 2013
4.	CpGLP1	Latex	Freitas et al., 2017
5.	CpGLP2	Latex	Freitas et al., 2017
6.	Procerain	Latex	Ramos et al., 2013
7.	Procerain B	Latex	Ramos et al., 2013
**Flavonoids**			
1.	3′-*O*-Methyl quercetin-3-*O*-rutinoside	Whole plant	Sweidan and Abu Zarga, 2015
2.	5-Hydroxy-3,7-dimethoxyflavone-4′-*O*-β-Glucopyranoside	Leaves	Nenaah, 2013b
3.	Isorhamnetin	Leaves	Nenaah, 2013b
4.	Kaempferol	Leaves	Nenaah, 2013b
5.	Rutin	Leaves	Nenaah, 2013b
**Lignans**			
1.	(+)-Pinoresinol 4-*O*-[6″-*O*-protocatechuoyl]-β-D-glucopyranoside	Latex	Abdel-Mageed et al., 2016
2.	(+)-Pinoresinol 4-*O*-[6″-*O*-vanillyl]-β-D-glucopyranoside	Latex	Abdel-Mageed et al., 2016
3.	(+)-Pinoresinol 4-*O*-β-D-glucopyranoside	Latex	Abdel-Mageed et al., 2016
4.	7′-Methoxy-3′-*O*-demethyl-tanegool-9-*O*-βD-glucopyranoside	Flower	Al-Taweel et al., 2017
5.	Eucommin A	Latex	Abdel-Mageed et al., 2016
6.	Pinoresinol-4′-*O*-[6″-*O*-(E)-feruloyl]-β-D-glucopyranoside	Latex	Abdel-Mageed et al., 2016
**Esters**			
1.	Calotropterpenyl ester	Root bark	Ansari and Ali, 2001
2.	Tridecyl ester	Leaves	Rani et al., 2019
**Volatiles**			
1.	1-Hexadecanol-2-methyl	Essential oil	Okiei et al., 2009
2.	1-Docosanol	Essential oil	Okiei et al., 2009
3.	1-Hexacosene	Leaves	Rani et al., 2019
4.	1-Nonadecene	Essential oil	Okiei et al., 2009
5.	2-Butanone-4,2,6,6-trimethyl-1-cyclohexen-1-yl	Essential oil	Okiei et al., 2009
6.	3,7,11,15-Tetramethyl-2-hexadecene-1-ol	Essential oil	Okiei et al., 2009
7.	3-Buten-2-one-4,2,6,6-trimethyl-1-cyclohexen-1-yl	Essential oil	Okiei et al., 2009
8.	4,8,12,16-Tetramethylheptadecan-4-olide	Essential oil	Okiei et al., 2009
9.	5,9,13-Pentadecatriene-2-one,6,10,14-trimethyl (E,E)	Essential oil	Okiei et al., 2009
10.	6,10,14-Trimethyl-2-pentadecanone	Essential oil	Okiei et al., 2009
11.	9,12-Octadecadienoyl chloride	Essential oil	Okiei et al., 2009
12.	9,17-Octadecadienal (Z)	Essential oil	Okiei et al., 2009
13.	9-Nonadecene	Essential oil	Okiei et al., 2009
14.	Hexadecanal	Essential oil	Okiei et al., 2009
15.	Isophytol	Essential oil	Okiei et al., 2009
16.	Mannosamine	Leaves	Rani et al., 2019
17.	Pentatriacontane	Leaves	Rani et al., 2019
18.	Phytol	Essential oil	Okiei et al., 2009
19.	R-Limonene	Leaves	Rani et al., 2019
20.	Tetradecanal	Essential oil	Okiei et al., 2009
21.	Tridecane	Leaves	Rani et al., 2019
22.	Z-5-Nonadecene	Essential oil	Okiei et al., 2009

Apart from that, the phytotoxicity of *C*. *procera* has also been tested against several crop and weed species. Aboveground plant extracts showed inhibition of seed germination and seedling growth in barley (*Hordeum vulgare* L.), wheat (*Triticum aestivum* L.), cucumber (*Cucumis sativus* L.), fenugreek (*Trigonella foenum-graecum* L.), tomato (*Solanum lycopersicum* L.), eggplant (*Solanum melongena* L.), lettuce (*Lactuca sativa* L.), *Senna occidentalis* (L.) Link, *Portulaca oleracea* L., *Chenopodium murale* L., *Pennisetum glaucum* (L.) R.Br., *Setaria italica* (L.) P.Beauv., and *Brassica rapa* L. (syn. *B*. *campestris*) (Hassan et al., 2015; Radwan et al., 2019; Al-Harbi, 2020; Hussain et al., 2020). Leaf, fruit, and flower extracts of *C*. *procera* significantly inhibited the germination, radicle length, plumule length, biomass accumulation, and relative water content in *Brassica cretica* Lam. (syn. *B*. *oleracea* var. *botrytis*) (Gulzar and Siddiqui, 2017). Similarly, essential oil of *C*. *procera* also showed potent phytotoxicity against *Bidens pilosa* L. and *Dactyloctenium aegyptium* (L.) Willd. (Al-Rowaily et al., 2020). Phytotoxic properties of *C*. *procera* may assist its establishment in non-native areas by negatively affecting the growth of resident vegetation. From an economic point of view, the phytotoxic potential of the weed can be exploited for the production of bioherbicides; however, more dose-response studies are required in this context.

## Economic Importance

### Pharmacological Applications

The search for environment-friendly prototypes to replace chemically synthesized drugs is rapidly increasing. Thus, a lot of research has been focused on the plant species mentioned in traditional medicinal systems. The pharmacological activities of *C*. *procera* have been popular in the past to cure several diseases in human beings such as cold, fever, leprosy, asthma, rheumatism, eczema, indigestion, diarrhea, elephantiasis, skin diseases, and dysentery (Al-Rowaily et al., 2020). The decoction of aboveground parts is being used to treat fever, joint pain, muscular spasm, and constipation in Saudi Arabia (Mossa et al., 1991). The plant is also used to treat neuropsychiatric disorders in Burkina Faso (Kinda et al., 2020). The medicinal attributes of *C*. *procera* can be credited to secondary metabolites and cardiotonic substances present in the plant (Hagaggi and Mohamed, 2020; Mehmood et al., 2020).

The extracts of aboveground plant parts of *C*. *procera* exhibited strong antipyretic, analgesic, antidepressant, and neuromuscular blocking activity (Mossa et al., 1991; Garabadu et al., 2019). Extracts from bark and leaves showed notable antibacterial potential against *Klebsiella pneumoniae*, *Pseudomonas aeruginosa*, *Bacillus subtilis*, and *Escherichia coli* (Mehmood et al., 2020). A broad antibacterial spectrum has been shown by extracts of both aerial parts of *C*. *procera* and its endophytic bacteria, *Bacillus siamensis* (Hagaggi and Mohamed, 2020). Leaf extracts of *C*. *procera* also reduce blood glucose to a significant level, thereby indicating its antihyperglycemic potential (Nadeem et al., 2019).

Latex of *C*. *procera* contains cardiac glycosides, which inhibit the proliferation of MCF-7 cells through cytotoxicity, apoptosis, and autophagy (Al-Qahtani et al., 2020). Chitinase isoforms present in the latex are also cytotoxic to tumor cell lines and are capable of reducing inflammation by iNOs-derived NO mechanism (Viana et al., 2017). Crude latex also possessed antioxidant and antiapoptotic activities against the toxicity of 4-Nonylphenol (Sayed et al., 2016). It has shown anthelmintic effects against *Haemonchus contortus* by damaging its cuticle and causing ultrastructural changes (Cavalcante et al., 2020). Latex of *C*. *procera* is also a promising phytotherapeutic option for treating inflammatory conditions of the colon (Kumar et al., 2019). The protein fraction of the latex has the potential to relieve inflammation and pain associated with arthritis (Kumar et al., 2011). Oral mucositis, an intense inflammatory reaction that can lead to tissue damage and ulceration, was found to be curable using PII-IAA, a homogenous cocktail of laticifer proteins of *C*. *procera* (Ramos et al., 2020). Similarly, intestinal mucositis is observed to be abolished by latex proteins of *C*. *procera* (de Alencar et al., 2017).

In addition to that, anti-inflammatory and gastromucosal protective effect of the stem bark of *C*. *procera* has also been observed (Tour and Talele, 2011). Root bark also consists of oxypregnane oligoglycosides, which has cytotoxic potential against U373 glioblastoma and PC-3 prostate cancer cell lines (Ibrahim et al., 2015). An earlier retrieval of sensorimotor activities, reduced ROS, increased total antioxidant activity (particularly, the enhanced activities of arylesterase and paraoxonase), suggested a positive impact of roots of *C*. *procera* on functional recovery upon a nerve injury (Zafar et al., 2020).

### Phytoremediation

*Calotropis procera* is a phytoaccumulator of several heavy metals such as manganese, lead, chromium, iron, copper, nickel, cobalt, strontium, and cadmium (D’Souza et al., 2010; Almehdi et al., 2019; Ullah and Muhammad, 2020). As determined from biophysical measurements, roots and leaves of *C*. *procera* are also tolerant against aluminum toxicity (Hussain et al., 2018). *C*. *procera* can also be used as a phytomonitoring tool to assess metals in the environment (Gajbhiye et al., 2019). A high accumulation of chromium has been observed in the roots (up to 188.2 mg kg^–1^) and shoots (up to 68.2 mg kg^–1^) of *C*. *procera*, which is detoxified by regulation of cellular homeostasis via redox signaling (Usman et al., 2020). Fruits and leaf powder of *C*. *procera* were also found to adsorb, respectively, Acid red 73 and Congo Red dye, the colorant dyes used in dyeing processes, which are harmful to aquatic life due to their release in the water bodies (Kaur and Kaur, 2017; EL-Adawy and Alomari, 2020). It has also been observed that old leaves of the plant have a greater ability to accumulate heavy metals compared to any other plant parts (Almehdi et al., 2019). This suggests that *C*. *procera* uses the metabolically less active leaves as sinks for heavy metals (Almehdi et al., 2019).

### Source of Fiber

*Calotropis procera* is an emerging source of natural fiber. Efforts have been put to screen efficient genotypes from its wild populations, which can be improved through conventional breeding programs to develop suitable varieties for cultivation (Majeed et al., 2020). Its fiber is natural, renewable with low density, high strength, crude oil sorption capacity (about 75 times its weight), and hydrophobic-oleophilic characteristics (Hilário et al., 2019; dos Anjos et al., 2020; Raghu and Goud, 2020). It is composed of 64.0 weight % cellulose, 19.5 weight % hemicelluloses, and 9.7 weight % of lignin (Song et al., 2019). The fibers exhibit thermal stability and can endure a temperature up to 200°C (Yoganandam et al., 2019). Alkali treatment may enhance the tensile strength, modulus, and length of the fiber (Raghu and Goud, 2020). The chemical polymerization of polyaniline enhances fiber conductivity (dos Santos et al., 2020). For increasing the absorption efficiency of organic oils and solvents, the fiber can be treated with 0.1 M sodium hydroxide or 1% sodium chlorite (dos Anjos et al., 2020). Also, fiber length can be improved by a cell expansion mechanism derived from plasma membrane intrinsic proteins (Aslam et al., 2013).

Owing to its antimicrobial tendency, the bast fiber from *C*. *procera* can substitute cotton (*Gossypium* sp.) wool for surgical or stuffing purposes (Basu, 2020). Stuffing material for mattresses and pillows can also be prepared from the fiber (Oun and Rhim, 2016). These natural fibers are also promising candidates for the fabrication of composites (Yoganandam et al., 2020) and the production of cellulose nanocrystals (Song et al., 2019). Reports suggest that fiber of *C*. *procera* can also be used as a biosorbent for the removal of contaminants due to oil spill (Hilário et al., 2019; dos Anjos et al., 2020).

### Synthesis of Nanoparticles

Green nanotechnology has become an emerging field for the cost-effective and eco-friendly production of metallic nanoparticles (NPs) for multiple industrial applications, and *C*. *procera* has successfully facilitated their fabrication. Cysteine proteases present in the latex were used to produce copper and gold NPs, which showed excellent biocompatibility with HeLa, A549, and BHK21 cell lines (Das et al., 2011; Harne et al., 2012). Silver NPs prepared using latex of *C*. *procera* showed strong antibacterial and antifungal activities (Mohamed et al., 2014). Cerium oxide NPs produced using *C*. *procera* flower extract have proved to be effective against gram-negative bacteria (Muthuvel et al., 2020). The therapeutic potential of silver NPs containing root extracts of *C*. *procera* was found to be significant against 10 strains of medically important bacteria and human epidermal primary keratinocytes cell line due to the metal-phytochemical moiety (Sagadevan et al., 2020). Similarly, iron NPs prepared in the leaf extracts are found to be efficient, cost-effective, and eco-friendly with strong antifungal activity (Ali et al., 2020a).

### Miscellaneous

*Calotropis procera* is used as an alternative for fodder during dry periods when other plant species are scarce (Frosi et al., 2013). Its use for fuel, timber, and building purposes dates back to the nineteenth century (Al Sulaibi et al., 2020; Batool et al., 2020). The plant has also been acknowledged for its ornamental value (de Oliveira et al., 2009). The plant yields valuable hydrocarbons and holds the potential to produce bioenergy and biofuel, which could be used as diesel substitutes in the future (Kumar, 2018). Studies also recommend the use of its enzyme extract to tenderize muscle foods such as pork, beef, and chicken (Rawdkuen et al., 2013), dehair crude leather (Lopéz et al., 2017), and coagulate milk for the production of fresh cheese (Abebe and Emire, 2020). *C*. *procera* leaves are also a potential source of natural colorants for textile fabrics (Hussaan et al., 2017). Cuticular wax derived from the plant is an eco-friendly hydrophobic material, which can have several industrial applications (Sharma et al., 2019). Apart from that, *C*. *procera* is one of the alternative raw materials for making excellent varieties of handmade paper (Aswal et al., 2020).

## *Calotropis procera* as an Invasive Species

*Calotropis procera* is a native of Asia and Africa but widely naturalized throughout the arid and semi-arid parts of the world (as described in section “Geographical Distribution”). Owing to its spread in new and larger areas, and adverse effects on the native ecosystems, *C*. *procera* has been declared as an invasive species in several regions of the world. It is a serious environmental weed of South America, the Caribbean Islands, Australia, the Hawaiian Islands, Mexico, Seychelles, and several Pacific Islands (Dhileepan, 2014).

In South America, the plant was introduced for ornamental and forage purposes; however, it has spread beyond the introduced areas by colonizing habitats with different environmental characteristics (Rivas et al., 2020). It is said to be an aggressive invader of the Caatinga ecoregion (Al Sulaibi et al., 2020) and others regions of northeastern Brazil where it has been introduced at the beginning of the nineteenth century (Frosi et al., 2013). The probability of its spread in the Canga ecoregion of Espinhaço mountain ranges of Brazil has also been suggested (de Oliveira et al., 2009). Due to its fast growth and drought tolerant abilities, it has spread extensively in the Caribbean Islands (Pompelli et al., 2019). Recently, it has been reported to spread along the coastal dunes of the Caribbean region of Colombia (Gracia et al., 2019).

In Australia, the plant may have introduced intentionally as an ornamental or accidentally with the packaging of camel saddles from India in the early 1900s (Dhileepan, 2014). It was reported from Katherine, Northern Territory, for the first time in the 1950s and thereafter, it has spread up to 3.7 million ha in drier parts of Northern Territory, Western Australia, and Queensland (Dhileepan, 2014). It has invaded the rangelands and Savannahs of Australia, threatening their biodiversity and productivity (Campbell et al., 2013, 2020). In the Gulf of Carpentaria region, its infestations have increased tremendously within the past few years and it has now approached the Burdekin catchment (Campbell et al., 2013). *C*. *procera* has also colonized in the rehabilitated Mary Kathleen uranium mine site in Queensland, Australia (Lottermoser, 2011). Ecological modeling based on climate change projections suggests that the uninvaded regions of northern and north-eastern territories of Western Australia and north-western Queensland are at potential risk of invasion by *C*. *procera* (Menge et al., 2016b).

*Calotropis procera* adopts an adult-persistence-population-survival strategy, characterized by lesser recruitment of fresh seedlings and relative stability of adult populations (Farahat et al., 2015). It can grow in a wide range of open habitats, such as along roadsides, watercourses, riverbeds, coastal dunes, deserts, semi-deserts, scrublands, overgrazed pastures, and disturbed areas (Dhileepan, 2014; Hassan et al., 2015). Being a metallophyte, *C*. *procera* invades polluted areas, contaminated sites, rehabilitated mines, ironstone rupestrian fields, etc., as pioneer vegetation (de Oliveira et al., 2009; Lottermoser, 2011). It also has a widespread persistence near unmanaged crop fields and thus, it may impose adverse effects on the crops through allelopathy (Hassan et al., 2015).

A phenological study of *C*. *procera* stated that ornamental and economic value of the plant leads to its distribution across the globe and functional traits such as large leaves, wind-dispersed seeds, hermaphrodite flowers, and ability to attract pollinators have facilitated its invasion process (Sobrinho et al., 2013). A difference in the reproductive phenology between the individuals of invaded range and native range has also been observed, with individuals present in the invaded range having a longer reproductive window (Sobrinho et al., 2013). Plasticity in phenological and functional attributes enables it to dominate the urban ecosystems of South Cairo, Egypt (Farahat et al., 2015; Pompelli et al., 2019). Disturbance levels in the soil also affect seed establishment in *C*. *procera*, and therefore, its uncontrolled spread is witnessed in areas subjected to natural and anthropogenic interference (Menge et al., 2017b). Also, *C*. *procera* is capable of defending itself against herbivores by producing latex with toxic steroidal cardenolides and releasing irritating volatiles (Fernandes et al., 2020).

Currently, management options practiced for *C*. *procera* include mechanical removal, chemical control, and management of invaded or susceptible areas. The plant can be removed mechanically along with its roots to prevent reproduction via suckers (Hassan et al., 2015). The use of mechanical equipment that severs the root system can achieve a mortality rate of up to 72% in *C*. *procera*, but the disturbance often promotes new seedling recruitments (Campbell et al., 2020). Foliar herbicides such as imazapyr, metsulfuron-methyl, 2,4-D butyl ester, fluroxypyr, triclopyr, and triclopyr plus picloram reported up to 80% efficacy in controlling the plants when applied to stump <5 cm in height (Vitelli et al., 2008). *C*. *procera* cannot stand competition with tall weeds, bushes, and grasses, and therefore, cannot invade intact grasslands (Menge et al., 2017b). Management is suggested in colder months when pollinator pressure is low and plants are not reproducing (Menge et al., 2017a). Because the plant needs nearly 1 year to produce fruits after emergence, conservation managers can manage its patch in a given area by constantly targeting new seedlings for 2 years to exhaust the seed bank (Bebawi et al., 2015).

In its native range, *C*. *procera* has several natural enemies that may act as potential biocontrol agents for the plant. A total of 65 insect species and five mite species have been reported to attack *C*. *procera* (Dhileepan, 2014). Among the herbivorous insects, larvae of *Danaus* spp. were observed to bypass host defenses, and feed on healthy, rapidly growing *C*. *procera* in the Brazilian Caatinga (Fernandes et al., 2020). The fruit fly, *Dacus persicus* Hendel (Diptera: Tephriti7dae) is also a prospective biological control agent for the plant in Australia owing to its field host specificity, high reproductive capacity, and damage potential (up to 100% damage to the immature seeds and 62% reduction in the biomass of infested fruits) (Ali et al., 2020b). Dhileepan (2014) suggested three pre-dispersal seed predators, *Paramecops farinosus* Schoenherr (Coleoptera: Curculionidae), *D*. *persicus* and *Dacus longistylus* Wiedemann (Diptera: Tephritidae) as prospective biocontrol agents of *C*. *procera*. Among the fungal pests, *Passalora calotropidis* is known to cause leaf spot disease in *C*. *procera* (Kiran et al., 2020). Since *C*. *procera* is an emerging invasive species of arid and semi-arid regions, suitable management strategies are needed to be devised and implemented as soon as possible so that spread and impact of the plant can be timely contained.

## Conclusions and Future Prospects

*Calotropis procera* is a plant with multifaceted biological characteristics that make it a medicinally and socio-economically important species on one hand and a potential invasive species on the other. The present discussion is meant to appraise its expanding global distribution, significant ecological and biological traits, applications in traditional and advanced fields, and infestation as an environmental weed. Also, it is an attempt to recognize the lesser-explored aspects and knowledge gaps in ongoing research.

Although pharmacological and industrial applications of the plant have received due attention, its general biological and ecological attributes (particularly those focusing on the adaptations or plasticity) have not been well-investigated. Also, the toxicity-bioactivity relationship of *C. procera*, which plays a key role in validating its medicinal aspects, has not been focused upon. Evaluating these basic facets may improve its commercial utilization and pave ways for novel applications. At the same time, covering these knowledge gaps can help understanding its invasive behavior and potential environmental or biodiversity threats that it can pose in the future.

In addition to that, the current and potential spread of *C. procera* is required to be mapped to carry out its timely management or containment, wherever required. The spread of *C. procera* can be effectively controlled in the invaded ranges via mechanical, chemical, or biological methods, followed by constant monitoring over the next few years to avoid new plantlets. Recognizing the plant as an important environmental weed can supplement its management programs at research, legislative, stakeholder, and local levels. Also, promoting its utilization at commercial and non-commercial scales can be an economically viable or better to say, economically beneficial way of its management.

## Author Contributions

DB and BC developed the initial concept and outline. AK and SK took lead in expanding the content. DB, SK, and BC contributed and edited the manuscript. All authors contributed to the article and approved the submitted version.

## Conflict of Interest

The authors declare that the research was conducted in the absence of any commercial or financial relationships that could be construed as a potential conflict of interest.

## References

[B1] Abdel-Mageed W. M. Mohamed N. H. Liu M. El-Gamal A. A. Basudan O. A. Ismail M. A. (2016). Lipoxygenase inhibitors from the latex of *Calotropis procera*. *Arch. Pharm. Res.* 10.1007/s12272-016-0725-9 [Epub ahead of print]. 26960736

[B2] Abebe B. Emire S. (2020). Manufacture of fresh cheese using east African *Calotropis procera* leaves extract crude enzyme as milk coagulant. *Food Sci. Nutr.* 8 4831–4842. 10.1002/fsn3.1765 32994945 PMC7500763

[B3] Al Sulaibi M. A. M. Thiemann C. Thiemann T. (2020). Chemical constituents and uses of *Calotropis procera* and *Calotropis gigantea*–a review (Part I–the plants as material and energy resources). *Open Chem. J.* 7 1–15. 10.2174/1874842202007010001

[B4] Al-Harbi N. A. (2020). Allelopathic effect of *Calotropis procera*, *Hyoscyamus muticus* and *Pulicaria undulata* extracts on seed germination of portulaca oleracea and chenopodium murale. *Pak. J. Biol. Sci.* 23 1260–1266. 10.3923/pjbs.2020.1260.1266 32981259

[B5] Ali M. Haroon U. Khizar M. Chaudhary H. J. Munis M. F. H. (2020a). Facile single step preparations of phyto-nanoparticles of iron in *Calotropis procera* leaf extract to evaluate their antifungal potential against *Alternaria alternata*. *Curr. Plant Biol.* 23:100157. 10.1016/j.cpb.2020.100157

[B6] Ali S. Shabbir A. Dhileepan K. (2020b). Bionomics and damage potential of fruit fly *Dacus persicus* (Diptera: Tephritidae): a prospective biological control agent of *Calotropis procera* (Apocynaceae). *Biocontrol Sci. Techn.* 30 716–727. 10.1080/09583157.2020.1765982

[B7] Almehdi A. El-Keblawy A. Shehadi I. El-Naggar M. Saadoun I. Mosa K. A. (2019). Old leaves accumulate more heavy metals than other parts of the desert shrub *Calotropis procera* at a traffic-polluted site as assessed by two analytical techniques. *Int. J. Phytoremediation* 21 1254–1262. 10.1080/15226514.2019.1619164 31134813

[B8] Al-Qahtani M. A. M. Farah M. A. Abou-Tarboush F. M. Al-Anazi K. M. Al-Harbi N. O. Ali M. A. (2020). Anticancer effects of *Calotropis procera* latex extract in MCF-7 breast cancer cells. *Pharmacogn. Mag.* 16 550–556. 10.4103/pm.pm_156_20

[B9] Al-Quwaie D. A. H. (2020). Bacterial community dynamics with rhizosphere of *Calotropis procera* and *Senna alexandrina* desert plants in Saudi Arabia. *Bioinformation* 16 567–578. 10.6026/97320630016567 33214744 PMC7649021

[B10] Al-Rowaily S. L. Abd-ElGawad A. M. Assaeed A. M. Elgamal A. M. El Gendy A. E. N. G. Mohamed T. A. (2020). Essential oil of *Calotropis procera*: comparative chemical profiles, antimicrobial activity, and allelopathic potential on weeds. *Molecules* 25:5203. 10.3390/molecules25215203 33182287 PMC7664932

[B11] Al-Taweel A. M. Perveen S. Fawzy G. A. Rehman A. U. Khan A. Mehmood R. (2017). Evaluation of antiulcer and cytotoxic potential of the leaf, flower, and fruit extracts of *Calotropis procera* and isolation of a new lignan glycoside. *Evid. Based Complement. Alternat. Med.* 2017:8086791. 10.1155/2017/8086791 28951762 PMC5603131

[B12] Ansari S. H. Ali M. (2001). Norditerpenic ester and pentacyclic triterpenoids from root bark of *Calotropis procera* (Ait) R. *Br. Pharmazie* 56 175–177. 11234349

[B13] Aslam U. Khatoon A. Cheema H. M. N. Basir A. (2013). Identification and characterization of plasma membrane aquaporins isolated from fiber cells of *Calotropis procera*. *J. Zhejiang Univ. Sci. B* 14 586–595. 10.1631/jzus.B1200233 23825144 PMC3709063

[B14] Aswal S. Chauhan S. Bhatnagar P. (2020). Identifying efficient isolates of white rot fungi for lignin degradation of *Calotropis procera* fibre in handmade papermaking. *J. Sci. Res.* 64 183–191. 10.37398/JSR.2020.640226

[B15] Basu A. (2020). Molecular docking study of expansin proteins in fibers of medicinal plants *Calotropis procera*. *Int. J. Appl. Res. Bioinform.* 10 10–17. 10.4018/IJARB.2020070102

[B16] Batool H. Hussain M. Hameed M. Ahmad R. (2020). A review on *Calotropis procera* its phytochemistry and traditional uses. *Big Data Agric.* 2 29–31. 10.26480/bda.02.2020.29.31

[B17] Bebawi F. F. Campbell S. D. Mayer R. J. (2015). Seed bank longevity and age to reproductive maturity of *Calotropis procera* (Aiton) WT Aiton in the dry tropics of northern Queensland. *Rangeland J.* 37 239–247. 10.1071/RJ14130

[B18] Bezerra C. F. Mota ÉF. Silva A. C. M. Tomé A. R. Silva M. Z. R. de Brito D. (2017). Latex proteins from *Calotropis procera*: toxicity and immunological tolerance revisited. *Chem. Biol. Interact.* 274 138–149. 10.1016/j.cbi.2017.07.007 28709944

[B19] CABI (2021). *Invasive Species Compendium.* Available online at: https://www.cabi.org/isc/datasheet/16848 (accessed January 6, 2021)

[B20] Campbell S. Roden L. Crowley C. (2013). “Calotrope (*Calotropis procera*): a weed on the move in northern Queensland,” in *Proceedings of the 12th Queensland Weed Symposium*, eds O’Brien M. Vitelli J. Thornby D. 11–14.

[B21] Campbell S. Roden L. O’Donnell C. Perkins M. (2020). The cutting depth required to control calotrope (*Calotropis procera*) plants using mechanical techniques. *Rangeland J.* 42 129–134. 10.1071/RJ20035

[B22] Cavalcante G. S. de Morais S. M. André W. P. P. de Araújo-Filho J. V. Muniz C. R. da Rocha L. O. (2020). Chemical constituents of *Calotropis procera* latex and ultrastructural effects on *Haemonchus contortus*. *Rev. Bras. Parasitol. Vet.* 29:e001320. 10.1590/s1984-29612020045

[B23] Chundattu S. J. Agrawal V. K. Ganesh N. (2016). Phytochemical investigation of *Calotropis procera*. *Arab. J. Chem.* 9 S230–S234. 10.1016/j.arabjc.2011.03.011

[B24] Coêlho M. R. V. Rivas R. Ferreira-Neto J. R. C. Bezerra-Neto J. P. Pandolfi V. Benko-Iseppon A. M. (2021). Salt tolerance of *Calotropis procera* begins with immediate regulation of aquaporin activity in the root system. *Physiol. Mol. Biol. Plants* 27 457–468. 10.1007/s12298-021-00957-9 33854276 PMC7981346

[B25] Das R. K. Sharma P. Nahar P. Bora U. (2011). Synthesis of gold nanoparticles using aqueous extract of *Calotropis procera* latex. *Mater. Lett.* 65 610–613. 10.1016/j.matlet.2010.11.040

[B26] de Alencar N. M. N. da Silveira Bitencourt F. de Figueiredo I. S. T. Luz P. B. Lima-Júnior R. C. P. Aragão K. S. (2017). Side-effects of irinotecan (CPT-11), the clinically used drug for colon cancer therapy, are eliminated in experimental animals treated with latex proteins from *Calotropis procera* (Apocynaceae). *Phytother. Res.* 31 312–320. 10.1002/ptr.5752 27910140

[B27] de Freitas C. D. T. de Souza Lopes J. L. Beltramini L. M. de Oliveira R. S. B. Oliveira J. T. A. Ramos M. V. (2011). Osmotin from *Calotropis procera* latex: new insights into structure and antifungal properties. *Biochim. Biophys. Acta Biomembranes* 1808 2501–2507. 10.1016/j.bbamem.2011.07.014 21798235

[B28] de Lima J. M. de Freitas F. J. C. Amorim R. N. L. Câmara A. C. L. Batista J. S. Soto-Blanco B. (2011). Clinical and pathological effects of *Calotropis procera* exposure in sheep and rats. *Toxicon* 57 183–185. 10.1016/j.toxicon.2010.11.007 21087619

[B29] de Oliveira S. H. F. Negreiros D. Fernandes G. W. Barbosa N. P. U. Rocha R. Almeida-Cortez J. S. (2009). Seedling growth of the invader *Calotropis procera* in ironstone rupestrian field and seasonally dry forest soils. *Neotropical Biol. Conserv.* 4 69–76. 10.4013/nbc.2009.42.01

[B30] Dhileepan K. (2014). Prospects for the classical biological control of *Calotropis procera* (Apocynaceae) using coevolved insects. *Biocontrol. Sci. Technol.* 24 977–998. 10.1080/09583157.2014.912611

[B31] dos Anjos R. B. Hilário L. S. de Moraes Juviniano H. B. da Silva D. R. (2020). Crude oil removal using *Calotropis procera*. *BioResources* 15 5246–5263.

[B32] dos Santos M. R. da Silva F. A. G. Jr. Ferrais P. P. de Lima R. S. da Costa M. M. de Oliveira H. P. (2020). Polyaniline-coated *Calotropis procera* L. hollow tubular fibers with remarkable antibacterial activity. *SN Appl. Sci.* 2:1550. 10.1007/s42452-020-03345-2

[B33] D’Souza R. J. Varun M. Masih J. Paul M. S. (2010). Identification of *Calotropis procera* L. as a potential phytoaccumulator of heavy metals from contaminated soils in Urban North Central India. *J. Hazard. Mater.* 184 457–464. 10.1016/j.jhazmat.2010.08.056 20843602

[B34] EL-Adawy H. A. Alomari A. A. (2020). Evaluation of *Calotropis procera* fruits as a bioadsorbent for removing of Acid Red 73 dye from the aqueous solutions. *Egypt. J. Chem.* 63 3217–3228. 10.21608/ejchem.2020.23834.2415

[B35] Farahat E. Galal T. El-Midany M. Hassan L. (2015). Effect of urban habitat heterogeneity on functional traits plasticity of the invasive species *Calotropis procera* (Aiton) W.T. *Aiton. Rend. Fis. Acc. Lincei* 26 193–201. 10.1007/s12210-015-0408-3

[B36] Fernandes G. W. de Almeida J. S. Rodrigues-Menelau M. F. V. Arantes-Garcia L. Novais S. (2020). The bigger the better? Vigour of the exotic host plant *Calotropis procera* (Apocynaceae) affects herbivory. *Neotropical. Biol. Conserv.* 15 359–366. 10.3897/neotropical.15.e55148

[B37] Freitas C. D. Freitas D. C. Cruz W. T. Porfírio C. T. M. N. Silva M. Z. R. Oliveira J. S. (2017). Identification and characterization of two germin-like proteins with oxalate oxidase activity from *Calotropis procera* latex. *Int. J. Biol. Macromol.* 105 1051–1061. 10.1016/j.ijbiomac.2017.07.133 28754622

[B38] Freitas C. D. Viana C. A. Vasconcelos I. M. Moreno F. B. B. Lima-Filho J. V. Oliveira H. D. (2016). First insights into the diversity and functional properties of chitinases of the latex of *Calotropis procera*. *Plant Physiol. Biochem.* 108 361–371. 10.1016/j.plaphy.2016.07.028 27521700

[B39] Freitas C. D. T. Oliveira J. S. Miranda M. R. A. Macedo N. M. R. Sales M. P. Villas-Boas L. A. (2007). Enzymatic activities and protein profile of latex from *Calotropis procera*. *Plant Physiol. Biochem.* 45 781–789. 10.1016/j.plaphy.2007.07.020 17888673

[B40] Freitas C. D. T. Silva R. O. Ramos M. V. Porfírio C. T. M. N. Farias D. F. Sousa J. S. (2020). Identification, characterization, and antifungal activity of cysteine peptidases from *Calotropis procera* latex. *Phytochemistry* 169:112163. 10.1016/j.phytochem.2019.112163 31605904

[B41] Frosi G. Oliveira M. T. Almeida-Cortez J. Santos M. G. (2013). Ecophysiological performance of *Calotropis procera*: an exotic and evergreen species in Caatinga, Brazilian semi-arid. *Acta Physiol. Plant.* 35 335–344. 10.1007/s11738-012-1076-x

[B42] Gajbhiye T. Pandey S. K. Lee S. S. Kim K. H. (2019). Size fractionated phytomonitoring of airborne particulate matter (PM) and speciation of PM bound toxic metals pollution through *Calotropis procera* in an urban environment. *Ecol. Indic.* 104 32–40. 10.1016/j.ecolind.2019.04.072

[B43] Garabadu D. Srivastava N. Murti Y. (2019). *Calotropis procera* attenuates chronic unpredictable mild stress-induced depression in experimental animals. *Metab. Brain Dis.* 34 1635–1647. 10.1007/s11011-019-00471-8 31346860

[B44] Gracia A. Rangel-Buitrago N. Castro-Barros J. D. (2019). Non-native plant species in the atlantico department coastal dune systems, caribbean of Colombia: a new management challenge. *Mar. Pollut. Bull.* 141 603–610. 10.1016/j.marpolbul.2019.03.009 30955773

[B45] GRIN (2021). *Plant Germplasm: Taxonomy.* Available online at: http://www.tn-grin.nat.tn/gringlobal/taxonomydetail.aspx?id=8653 (accessed January 6, 2021)

[B46] Gulzar A. Siddiqui M. B. (2017). Allelopathic effect of *Calotropis procera* (Ait.) R. Br. on growth and antioxidant activity of *Brassica oleracea* var. botrytis. *J. Saudi Soc. Agric. Sci.* 16 375–382. 10.1016/j.jssas.2015.12.003

[B47] Hagaggi N. S. A. Mohamed A. A. A. (2020). Plant–bacterial endophyte secondary metabolite matching: a case study. *Arch. Microbiol.* 202 2679–2687. 10.1007/s00203-020-01989-7 32719949

[B48] Harne S. Sharma A. Dhaygude M. Joglekar S. Kodam K. Hudlikar M. (2012). Novel route for rapid biosynthesis of copper nanoparticles using aqueous extract of *Calotropis procera* L. latex and their cytotoxicity on tumor cells. *Colloids Surf. B: Biointerfaces* 95 284–288. 10.1016/j.colsurfb.2012.03.005 22483347

[B49] Hassan L. M. Galal T. M. Farahat E. A. El-Midany M. M. (2015). The biology of *Calotropis procera* (Aiton) WT. *Trees* 29 311–320. 10.1007/s00468-015-1158-7

[B50] Hilário L. S. dos Anjos R. B. de Moraes Juviniano H. B. da Silva D. R. (2019). Evaluation of thermally treated *Calotropis procera* fiber for the removal of crude oil on the water surface. *Materials* 12:3894. 10.3390/ma12233894 31775373 PMC6926797

[B51] Hussaan M. Iqbal N. Adeel S. Azeem M. Javed M. T. Raza A. (2017). Microwave-assisted enhancement of milkweed (*Calotropis procera* L.) leaves as an eco-friendly source of natural colorants for textile. *Environ. Sci. Pollut. Res.* 24 5089–5094. 10.1007/s11356-016-8162-3 27988899

[B52] Hussain F. Rasool A. Aziz K. Raisham S. Aziz S. Badshah L. (2020). Allelopathic inhibition of germination, seedling growth and cell division of selected plant species by *Calotropis procera* (Ait.) Ait. *Plant Sci. Today* 7 1–8. 10.14719/pst.2020.7.1.606

[B53] Hussain M. I. El-Keblawy A. Elwakil A. S. (2018). Aluminum influence on *Calotropis procera* seedling growth, nutrient accumulation and electrochemical attributes. *Flora* 248 34–42. 10.1016/j.flora.2018.08.012

[B54] Ibrahim S. R. M. Mohamed G. A. Shaala L. A. Banuls L. M. Y. Kiss R. Youssef D. T. A. (2015). Calotroposides H–N, new cytotoxic oxypregnane oligoglycosides from the root bark of *Calotropis procera*. *Steroids* 96 63–72. 10.1016/j.steroids.2015.01.012 25641077

[B55] Ibrahim S. R. M. Mohamed G. A. Shaala L. A. Banuls L. M. Y. Van Goietsenoven G. Kiss R. (2012). New ursane-type triterpenes from the root bark of *Calotropis procera*. *Phytochem. Lett.* 5 490–495. 10.1016/j.phytol.2012.04.012

[B56] Kakkar A. Verma D. R. Suryavanshi S. Dubey P. (2012). Characterization of chemical constituents of *Calotropis procera*. *Chem. Nat. Compd.* 48 155–157. 10.1007/s10600-012-0189-1

[B57] Kaur R. Kaur H. (2017). *Calotropis procera* an effective adsorbent for removal of Congo red dye: isotherm and kinetics modelling. *Model. Earth Syst. Environ.* 3:9. 10.1007/s40808-017-0274-3

[B58] Khalid N. Noman A. Sanaullah T. Akram M. A. Aqeel M. (2018). Vehicle pollution toxicity induced changes in physiology, defence system and biochemical characteristics of *Calotropis procera* L. *Chem. Ecol.* 34 565–581. 10.1080/02757540.2018.1452917

[B59] Khan A. Nasreen N. Niaz S. Ayaz S. Naeem H. Muhammad I. (2019). Acaricidal efficacy of *Calotropis procera* (Asclepiadaceae) and taraxacum officinale (Asteraceae) against *Rhipicephalus microplus* from Mardan, Pakistan. *Exp. Appl. Acarol.* 78 595–608. 10.1007/s10493-019-00406-z 31367977

[B60] Kinda P. T. Nacoulma A. P. Guenné S. Compaoré M. Djandé A. Lagnika L. (2020). The metabolomic study of *Calotropis procera* Ait. from Burkina Faso, based on chemical functional groups profiling using FTIR. *J. Complement. Integr. Med.* 17:20190134. 10.1515/jcim-2019-0134 32543456

[B61] Kiran K. Mahey S. Sharma A. Kumar V. Sharma A. Arora S. (2020). Post-infectional changes associated with the progression of leaf spot disease in *Calotropis procera* Aiton. *Physiol. Mol. Plant Pathol.* 112:101519. 10.1016/j.pmpp.2020.101519

[B62] Kumar A. (2018). “Alternative biomass from semiarid and arid conditions as a biofuel source: *Calotropis procera* and its genomic characterization,” in *Biofuels: Greenhouse Gas Mitigation and Global Warming*, eds Kumar A. Ogita S. Yau Y. Y. (New Delhi: Springer), 241–269. 10.1007/978-81-322-3763-1_14

[B63] Kumar V. L. Chaudhary P. Ramos M. V. Mohan M. Matos M. P. (2011). Protective effect of proteins derived from the latex of *Calotropis procera* against inflammatory hyperalgesia in monoarthritic rats. *Phytother. Res.* 25 1336–1341. 10.1002/ptr.3428 21328619

[B64] Kumar V. L. Pandey A. Verma S. Das P. (2019). Protection afforded by methanol extract of *Calotropis procera* latex in experimental model of colitis is mediated through inhibition of oxidative stress and pro-inflammatory signaling. *Biomed. Pharmacother.* 109 1602–1609. 10.1016/j.biopha.2018.10.187 30551414

[B65] Lopéz L. M. I. Viana C. A. Errasti M. E. Garro M. L. Martegani J. E. Mazzilli G. A. (2017). Latex peptidases of *Calotropis procera* for dehairing of leather as an alternative to environmentally toxic sodium sulfide treatment. *Bioprocess Biosyst. Eng.* 40 1391–1398. 10.1007/s00449-017-1796-9 28624929

[B66] Lottermoser B. G. (2011). Colonisation of the rehabilitated mary kathleen uranium mine site (Australia) by *Calotropis procera*: toxicity risk to grazing animals. *J. Geochem. Explor.* 111 39–46. 10.1016/j.gexplo.2011.07.005

[B67] Majeed A. Goel B. Mishra V. Kohli R. Bhardwaj P. (2020). Elucidation of genetic diversity base in *Calotropis procera*–a potentially emerging new fibre resource. *Plant Genet. Resour.* 18 159–167. 10.1017/S1479262120000167

[B68] Mehmood T. Arshad H. Nawaz S. Ullah A. Hafeez A. Anwar F. (2020). Pharmaceutical potential and phenolics profiling of leaves and bark of *Calotropis procera* in relation to extraction solvents. *Pharm. Chem. J.* 54 631–641. 10.1007/s11094-020-02250-7

[B69] Menge E. O. Bellairs S. M. Lawes M. J. (2016a). Seed-germination responses of *Calotropis procera* (Asclepiadaceae) to temperature and water stress in northern Australia. *Aust. J. Bot.* 64 441–450. 10.1071/BT16044

[B70] Menge E. O. Bellairs S. M. Lawes M. J. (2017b). Disturbance-dependent invasion of the woody weed, *Calotropis procera*, in Australian rangelands. *Rangeland J.* 39 201–211. 10.1071/RJ16120

[B71] Menge E. O. Greenfield M. L. McConchie C. A. Bellairs S. M. Lawes M. J. (2017a). Density-dependent reproduction and pollen limitation in an invasive milkweed, *Calotropis procera* (Ait.) R. Br.(Apocynaceae). *Austral Ecol.* 42 61–71. 10.1111/aec.12401

[B72] Menge E. O. Stobo-Wilson A. Oliveira S. L. J. Lawes M. J. (2016b). The potential distribution of the woody weed *Calotropis procera* (Aiton) WT Aiton (Asclepiadaceae) in Australia. *Rangeland J.* 38 35–46. 10.1071/RJ15081

[B73] Mittal A. Ali M. (2015). Acyclic diterpenic constituents from the roots of *Calotropis procera* (Ait.) R. *Br. J. Saudi Chem. Soc.* 19 59–63. 10.1016/j.jscs.2011.12.019

[B74] Mohamed N. H. Ismail M. A. Abdel-Mageed W. M. Shoreit A. A. M. (2014). Antimicrobial activity of latex silver nanoparticles using *Calotropis procera*. *Asian Pac. J. Trop. Biomed.* 4 876–883. 10.12980/APJTB.4.201414B216

[B75] Mohamed N. H. Liu M. Abdel-Mageed W. M. Alwahibi L. H. Dai H. Ismail M. A. (2015). Cytotoxic cardenolides from the latex of *Calotropis procera*. *Bioorg. Med. Chem. Lett.* 25 4615–4620. 10.1016/j.bmcl.2015.08.044 26323871

[B76] Mossa J. S. Tariq M. Mohsin A. Ageel A. M. Al-Yahya M. A. Al-Said M. S. (1991). Pharmacological studies on aerial parts of *Calotropis procera*. *Am. J. Chinese Med.* 19 223–231. 10.1142/S0192415X91000302 1767794

[B77] Moustafa A. M. Y. Ahmed S. H. Nabil Z. I. Hussein A. A. Omran M. A. (2010). Extraction and phytochemical investigation of *Calotropis procera*: effect of plant extracts on the activity of diverse muscles. *Pharm. Biol.* 48 1080–1190. 10.3109/13880200903490513 20690894

[B78] Muthuvel A. Jothibas M. Mohana V. Manoharan C. (2020). Green synthesis of cerium oxide nanoparticles using *Calotropis procera* flower extract and their photocatalytic degradation and antibacterial activity. *Inorg. Chem. Commun.* 119:108086. 10.1016/j.inoche.2020.108086

[B79] Mutwakil M. Z. Hajrah N. H. Atef A. Edris S. Sabir M. J. Al-Ghamdi A. K. (2017). Transcriptomic and metabolic responses of *Calotropis procera* to salt and drought stress. *BMC Plant Biol.* 17:231. 10.1186/s12870-017-1155-7 29202709 PMC5716246

[B80] Nadeem M. Mumtaz M. W. Danish M. Rashid U. Mukhtar H. Anwar F. (2019). *Calotropis procera*: UHPLC-QTOF-MS/MS based profiling of bioactives, antioxidant and anti-diabetic potential of leaf extracts and an insight into molecular docking. *J. Food Meas. Charact.* 13 3206–3220. 10.1007/s11694-019-00243-z

[B81] Nascimento T. L. Oki Y. Lima D. M. M. Almeida-Cortez J. S. Fernandes G. W. Souza-Motta C. M. (2015). Biodiversity of endophytic fungi in different leaf ages of *Calotropis procera* and their antimicrobial activity. *Fungal Ecol.* 14 79–86. 10.1016/j.funeco.2014.10.004

[B82] Nenaah G. (2013b). Antimicrobial activity of *Calotropis procera* Ait. (Asclepiadaceae) and isolation of four flavonoid glycosides as the active constituents. *World J. Microbiol. Biotechnol.* 29 1255–1262. 10.1007/s11274-013-1288-2 23417281

[B83] Nenaah G. E. (2013a). Potential of using flavonoids, latex and extracts from *Calotropis procera* (Ait.) as grain protectants against two coleopteran pests of stored rice. *Ind. Crops Prod.* 45 327–334. 10.1016/j.indcrop.2012.12.043

[B84] Okiei W. Ogunlesi M. Ofor E. Osibote E. A. S. (2009). Analysis of essential oil constituents in hydro-distillates of *Calotropis procera* (Ait.) R.Br. *Res J. Phytochem.* 2009 1–10.

[B85] Oun A. A. Rhim J. W. (2016). Characterization of nanocelluloses isolated from Ushar (*Calotropis procera*) seed fiber: Effect of isolation method. *Mater. Lett.* 168 146–150. 10.1016/j.matlet.2016.01.052

[B86] Pattnaik P. K. Kar D. Chhatoi H. Shahbazi S. Ghosh G. Kuanar A. (2017). Chemometric profile & antimicrobial activities of leaf extract of *Calotropis procera* and *Calotropis gigantea*. *Nat. Prod. Res.* 31 1954–1957. 10.1080/14786419.2016.1266349 27936921

[B87] Pompelli M. F. Mendes K. R. Ramos M. V. Santos J. N. B. Youssef D. T. A. Pereira J. D. (2019). Mesophyll thickness and sclerophylly among *Calotropis procera* morphotypes reveal water-saved adaptation to environments. *J. Arid Land* 11 795–810. 10.1007/s40333-019-0016-7

[B88] Radwan A. M. Alghamdi H. A. Kenawy S. K. M. (2019). Effect of *Calotropis procera* L. plant extract on seeds germination and the growth of microorganisms. *Ann. Agric. Sci.* 64 183–187. 10.1016/j.aoas.2019.12.001

[B89] Raghu M. J. Goud G. (2020). Effect of surface treatment on mechanical properties of *Calotropis procera* natural fiber reinforced epoxy polymer composites. *AIP Conf. Proc.* 2274:030031. 10.1063/5.0022584

[B90] Ramadan A. Sabir J. S. M. Alakilli S. Y. M. Shokry A. M. Gadalla N. O. Edris S. (2014). Metabolomic response of *Calotropis procera* growing in the desert to changes in water availability. *PLoS One* 9:e87895. 10.1371/journal.pone.0087895 24520340 PMC3919747

[B91] Ramos M. V. Araújo E. S. Jucá T. L. Monteiro-Moreira A. C. O. Vasconcelos I. M. Moreira R. A. (2013). New insights into the complex mixture of latex cysteine peptidases in *Calotropis procera*. *Int. J. Biol. Macromol.* 58 211–219. 10.1016/j.ijbiomac.2013.04.001 23583491

[B92] Ramos M. V. Freitas A. P. F. Leitão R. F. C. Costa D. V. S. Cerqueira G. S. Martins D. S. (2020). Anti inflammatory latex proteins of the medicinal plant *Calotropis procera*: a promising alternative for oral mucositis treatment. *Inflamm. Res.* 69 951–966. 10.1007/s00011-020-01365-7 32488316

[B93] Rani R. Sharma D. Chaturvedi M. Yadav J. P. (2017). Antibacterial activity of twenty different endophytic fungi isolated from *Calotropis procera* and time kill assay. *Clin. Microbiol.* 6:1000280. 10.4172/2327-5073.1000280

[B94] Rani R. Sharma D. Chaturvedi M. Yadav J. P. (2019). Phytochemical analysis, antibacterial and antioxidant activity of *Calotropis procera* and *Calotropis gigantea*. *Nat. Prod. J.* 9 47–60. 10.2174/2210315508666180608081407

[B95] Rawdkuen S. Jaimakreu M. Benjakul S. (2013). Physicochemical properties and tenderness of meat samples using proteolytic extract from *Calotropis procera* latex. *Food Chem.* 136 909–916. 10.1016/j.foodchem.2012.08.077 23122144

[B96] Rivas R. Barros V. Falcão H. Frosi G. Arruda E. Santos M. (2020). Ecophysiological traits of invasive C3 species *Calotropis procera* to maintain high photosynthetic performance under high VPD and low soil water balance in semi-arid and seacoast zones. *Front. Plant Sci.* 11:717. 10.3389/fpls.2020.00717 32714338 PMC7343903

[B97] Rivas R. Frosi G. Ramos D. G. Pereira S. Benko-Iseppon A. M. Santos M. G. (2017). Photosynthetic limitation and mechanisms of photoprotection under drought and recovery of *Calotropis procera*, an evergreen C_3_ from arid regions. *Plant Physiol. Biochem.* 118 589–599. 10.1016/j.plaphy.2017.07.026 28793281

[B98] Sagadevan S. Vennila S. Muthukrishnan L. Gurunathan K. Oh W. C. Paiman S. (2020). Exploring the therapeutic potentials of phyto-mediated silver nanoparticles formed via *Calotropis procera* (Ait.) R. Br. Root Extract. *J. Exp. Nanosci.* 15 217–232. 10.1080/17458080.2020.1769842

[B99] Sayed A. D. H. Mohamed N. H. Ismail M. A. Abdel-Mageed W. M. Shoreit A. A. M. (2016). Antioxidant and antiapoptotic activities of *Calotropis procera* latex on Catfish (*Clarias gariepinus*) exposed to toxic 4-nonylphenol. *Ecotoxicol. Environ. Saf.* 128 189–194. 10.1016/j.ecoenv.2016.02.023 26946283

[B100] Sharma P. Madhyastha H. Madhyastha R. Nakajima Y. Maruyama M. Verma K. S. (2019). An appraisal of cuticular wax of *Calotropis procera* (Ait.) R. Br.: Extraction, chemical composition, biosafety and application. *J. Hazard. Mater.* 368 397–403. 10.1016/j.jhazmat.2019.01.067 30690392

[B101] Sobrinho M. S. Tabatinga G. M. Machado I. C. Lopes A. V. (2013). Reproductive phenological pattern of *Calotropis procera* (Apocynaceae), an invasive species in Brazil: annual in native areas; continuous in invaded areas of Caatinga. *Acta Bot. Brasilica* 27 456–459. 10.1590/S0102-33062013000200018

[B102] Song K. Zhu X. Zhu W. Li X. (2019). Preparation and characterization of cellulose nanocrystal extracted from *Calotropis procera* biomass. *Bioresour. Bioprocess.* 6:45. 10.1186/s40643-019-0279-z

[B103] Sweidan N. I. Abu Zarga M. H. (2015). Two novel cardenolides from *Calotropis procera*. *J. Asian Nat. Prod. Res.* 17 900–907. 10.1080/10286020.2015.1040772 25971597

[B104] Tezara W. Colombo R. Coronel I. Marín O. (2011). Water relations and photosynthetic capacity of two species of Calotropis in a tropical semi-arid ecosystem. *Ann. Bot.* 107 397–405. 10.1093/aob/mcq245 21149276 PMC3043925

[B105] Tour N. Talele G. (2011). Anti-inflammatory and gastromucosal protective effects of *Calotropis procera* (Asclepiadaceae) stem bark. *J. Nat. Med.* 65 598–605. 10.1007/s11418-011-0522-1 21400248

[B106] Ullah R. Muhammad S. (2020). Heavy metals contamination in soils and plants along with the mafic-ultramafic complex (Ophiolites), Baluchistan, Pakistan: Evaluation for the risk and phytoremediation potential. *Environ. Technol. Innov.* 19:100931. 10.1016/j.eti.2020.100931

[B107] Usman K. Al Jabri H. Abu-Dieyeh M. H. Alsafran M. H. S. A. (2020). Comparative assessment of toxic metals bioaccumulation and the mechanisms of chromium (Cr) tolerance and uptake in *Calotropis procera*. *Front. Plant Sci.* 11:883. 10.3389/fpls.2020.00883 32636868 PMC7317033

[B108] Viana C. A. Ramos M. V. Filho J. D. B. M. Lotufo L. V. C. Figueiredo I. S. T. de Oliveira J. S. (2017). Cytotoxicity against tumor cell lines and anti-inflammatory properties of chitinases from *Calotropis procera* latex. *Naunyn Schmiedebergs Arch. Pharmacol.* 390 1005–1013. 10.1007/s00210-017-1397-9 28698893

[B109] Vitelli J. Madigan B. Wilkinson P. van Haaren P. (2008). Calotrope (*Calotropis procera*) control. *Rangeland J.* 30 339–348. 10.1071/RJ07064

[B110] Yoganandam K. Ganeshan P. NagarajaGanesh B. Raja K. (2020). Characterization studies on *Calotropis procera* fibers and their performance as reinforcements in epoxy matrix. *J. Nat. Fibers* 17 1706–1718. 10.1080/15440478.2019.1588831

[B111] Yoganandam K. NagarajaGanesh B. Ganeshan P. Raja K. (2019). Thermogravimetric analysis of *Calotropis procera* fibers and their influence on the thermal conductivity and flammability studies of polymer composites. *Mater. Res. Express* 6:105341. 10.1088/2053-1591/ab3bbe

[B112] Zafar S. Anwar H. Qasim M. Irfan S. Maqbool J. Sajid F. (2020). *Calotropis procera* (root) escalates functions rehabilitation and attenuates oxidative stress in a mouse model of peripheral nerve injury. *Pak. J. Pharm. Sci.* 33 2801–2807. 10.36721/PJPS.2020.33.6.SUP.2801-2807.1 33879440

