# Supplementary figures and images for: An Overview of the Characteristics and Potential of Calotropis procera From Botanical, Ecological, and Economic Perspectives
Source: Front Plant Sci. 2021 Jun 17;12:690806. doi: 10.3389/fpls.2021.690806 (PMC8248367; doi:10.3389/fpls.2021.690806)

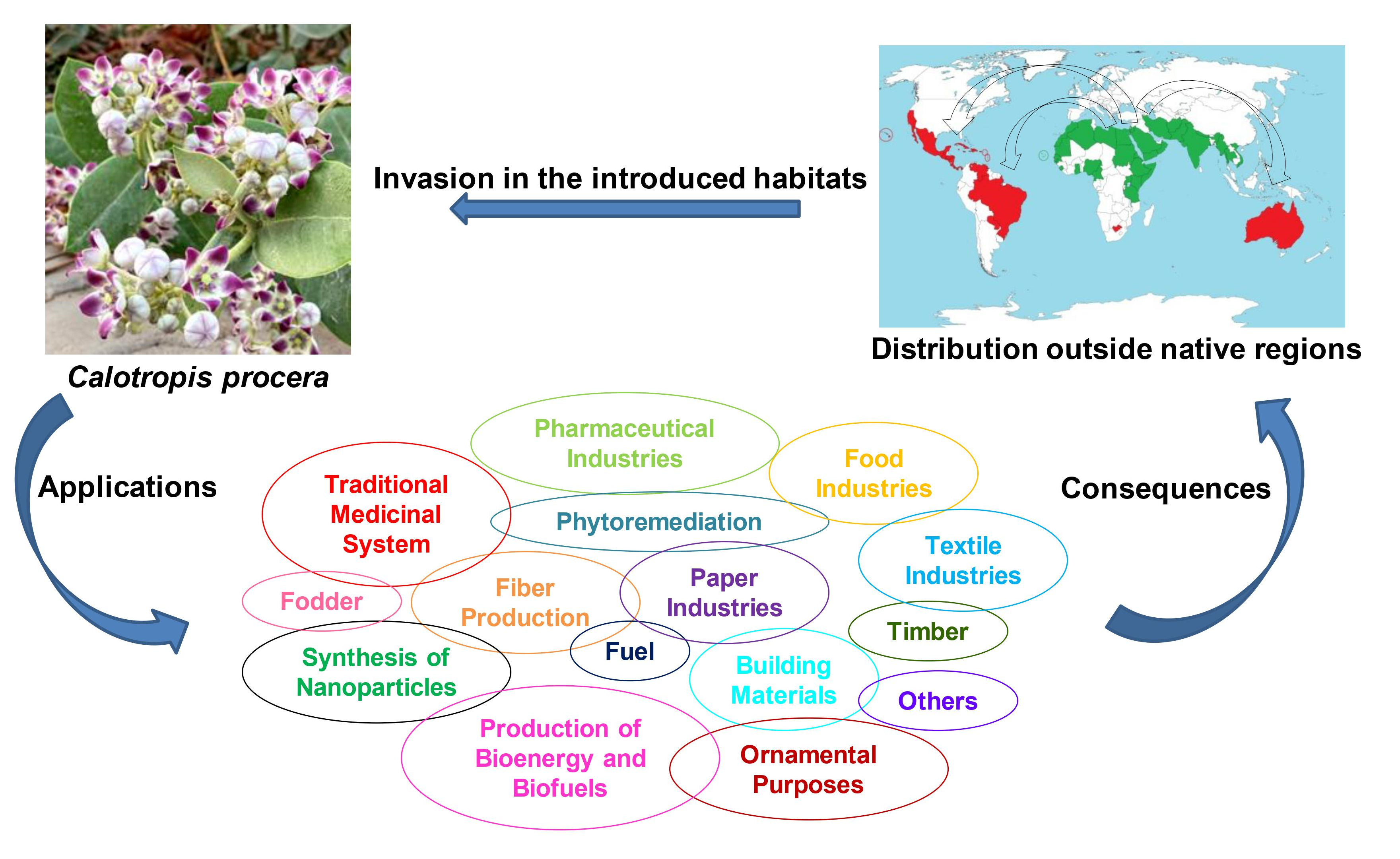

Supplement: Supplementary file 1 [file Image_1.TIF]
